# High-Power Electromagnetic Pulse Exposure of Healthy Mice: Assessment of Effects on Mice Cognitions, Neuronal Activities, and Hippocampal Structures

**DOI:** 10.3389/fncel.2022.898164

**Published:** 2022-06-29

**Authors:** Yanhui Hao, Weiqi Liu, Zhengtao Xu, Xing Jin, Yumeng Ye, Chao Yu, Cuicui Hu, Hongyan Zuo, Yang Li

**Affiliations:** ^1^Department of Experimental Pathology, Beijing Institute of Radiation Medicine, Beijing, China; ^2^Life Science Department, Foshan University, Foshan, China; ^3^Academy of Life Sciences, Anhui Medical University, Hefei, China

**Keywords:** electromagnetic pulse, mouse, cognition, neuron, calcium

## Abstract

Electromagnetic pulse (EMP) is a high-energy pulse with an extremely rapid rise time and a broad bandwidth. The brain is a target organ sensitive to electromagnetic radiation (EMR), the biological effects and related mechanisms of EMPs on the brain remain unclear. The objectives of the study were to assess the effects of EMP exposure on mouse cognitions, and the neuronal calcium activities *in vivo* under different cases of real-time exposure and post exposure. EMP-treated animal model was established by exposing male adult C57BL/6N mice to 300 kV/m EMPs. First, the effects of EMPs on the cognitions, including the spatial learning and memory, avoidance learning and memory, novelty-seeking behavior, and anxiety, were assessed by multiple behavioral experiments. Then, the changes in the neuronal activities of the hippocampal CA1 area *in vivo* were detected by fiber photometry in both cases of during real-time EMP radiation and post-exposure. Finally, the structures of neurons in hippocampi were observed by optical microscope and transmission electron microscope. We found that EMPs under this condition caused a decline in the spatial learning and memory ability in mice, but no effects on the avoidance learning and memory, novelty-seeking behavior, and anxiety. The neuron activities of hippocampal CA1 were disturbed by EMP exposure, which were inhibited during EMP exposure, but activated immediately after exposure end. Additionally, the CA1 neuron activities, when mice entered the central area in an Open field (OF) test or explored the novelty in a Novel object exploration (NOE) test, were inhibited on day 1 and day 7 after radiation. Besides, damaged structures in hippocampal neurons were observed after EMP radiation. In conclusion, EMP radiation impaired the spatial learning and memory ability and disturbed the neuronal activities in hippocampal CA1 in mice.

## Introduction

As a special electromagnetic phenomenon, electromagnetic pulses (EMPs) are high-energy, nonionizing radiation with a rapid rise time and a wide spectrum. Generally, EMP exposure mainly comes from the occupational environment, especially the operators who conduct electromagnetic damage resistance tests on electronic components. The potential health hazards caused by EMP exposure have received increasing attention, and no relevant medical protection standards have been established in China or elsewhere (Giri and Tesche, 2004; Kolosnjaj-Tabi et al., 2021). There are studies found that a certain dose of EMP causes the opening of the blood-brain barrier, which is a potential medication strategy for central nervous system diseases (Ding et al., 2010; Qiu et al., 2010; Zhou et al., 2013, 2017; Li et al., 2018; Gao et al., 2020; Wang et al., 2021). Therefore, study on the biological effects of EMPs can help in understanding its potential health hazards to the human body, and exploring the clinical application potential of EMPs, which is of great significance for the better utilization of this technology.

The brain is recognized as a target organ sensitive to electromagnetic radiation (EMR; Shahin et al., 2018; Narayanan et al., 2019; Gökçek-Saraç et al., 2021; Hu et al., 2021; Tan et al., 2021). The toxic effects of EMR are often classified as thermal or nonthermal effects. Radiofrequency (RF) radiation (100 kHz–300 GHz) is the most widely used type of EMR, of which the effects on organisms are mainly achieved by thermal effects, and the nonthermal effects are still controversial [International Commission on Non-Ionizing Radiation Protection (ICNIRP), (2020)]. As a special type of EMR, EMPs have relatively large energy that exists over a very short period of time, but cause no obvious thermal effects due to a low duty cycle. Therefore, EMPs may have different biological effects and mechanisms than radiofrequency radiation. Some studies have found that EMPs can cause damages to the cognitive functions, destruction of the blood-brain barrier, and alterations in the inflammatory responses (Qiu et al., 2010, 2011; Jiang et al., 2013, 2016; Kolosnjaj-Tabi et al., 2021). Overall, very few literatures in this field can be retrieved, leaving the researches on the biological effects of EMPs on the brain and the underlying mechanisms very unsatisfactory.

In this study, male C57BL/6N mice were used as the study subjects to evaluate the effects of EMPs on spatial learning and memory, working memory, active avoidance response, and anxiety by using a variety of behavioral methods. Combined with genetically encoded calcium indicator GCaMP, the fiber photometry technique can detect changes in calcium activities at the level of neurons *in vivo*. Currently, this technique has not been reported to be applied in the study of the neurobiological effects mediated by EMR. Through fiber photometry, the changes in the calcium activity of neurons in the hippocampal CA1 area were detected in both cases of real-time exposure to EMPs and post-exposure. Finally, changes in the structures in hippocampi after EMP radiation were observed. This study aimed to clarify the effects of EMPs on the cognitive function of the brain and the patterns of the changes in the neuron activities induced by EMP radiation *in vivo*.

## Materials and Methods

### Animals

All animal experimental procedures were performed at the Beijing Institute of Radiation Medicine, and were approved by the Institutional Animal Care and Use Committee of the Beijing Institute of Radiation Medicine, and were in accordance with the National Institutes of Health Guide for the Care and Use of Laboratory Animals.

A total of 165 male C57BL/6N mice (8-week-old, specific pathogen-free) were used in the study, which were provided by Weitonglihua Co., Ltd. (Beijing, China). All animals were maintained in the Laboratory Animal Center of Beijing Institute of Radiation Medicine, where the temperature was 22°C ± 2°C and the humidity 55% ± 5% on a 12 h light-dark cycle. Food and water were freely available. The mice were randomly divided into two groups: the control (Con), and the 300 kV/m EMP-exposed (EMP).

### Experimental Design Timeline

**Figure 1:**
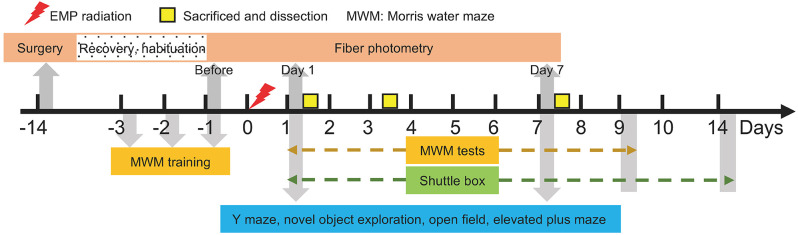
Temporal design of the experiments.

The temporal design is presented in Figure 1. The EMP radiation was applied on day 0. Morris water maze training was carried out from day 3 to day 1 ahead of EMP exposure, and the probe tests were performed from day 1 to day 9 after radiation. Shuttle box tests were performed from day 1 to day 14 after radiation. The other behavioral experiments, including Y maze, novel object exploration, open field and elevated plus maze, were applied on day 1 and day 7 after radiation. To avoid mutual interference, different animals were used to complete different behavioral experiments. As for fiber photometry, the surgery was performed 14 days ahead of data recording (day 14) to allow recovery and habituation, and the data were collected 5 min before, during and 5 min after the EMP radiation, or on day 1, day 1, and day 7 in another case. On day 1, day 3, and day 7 after radiation, the mice were anesthetized and decapitated for pathology tests.

### The EMP Exposure System

**Figure 2:**
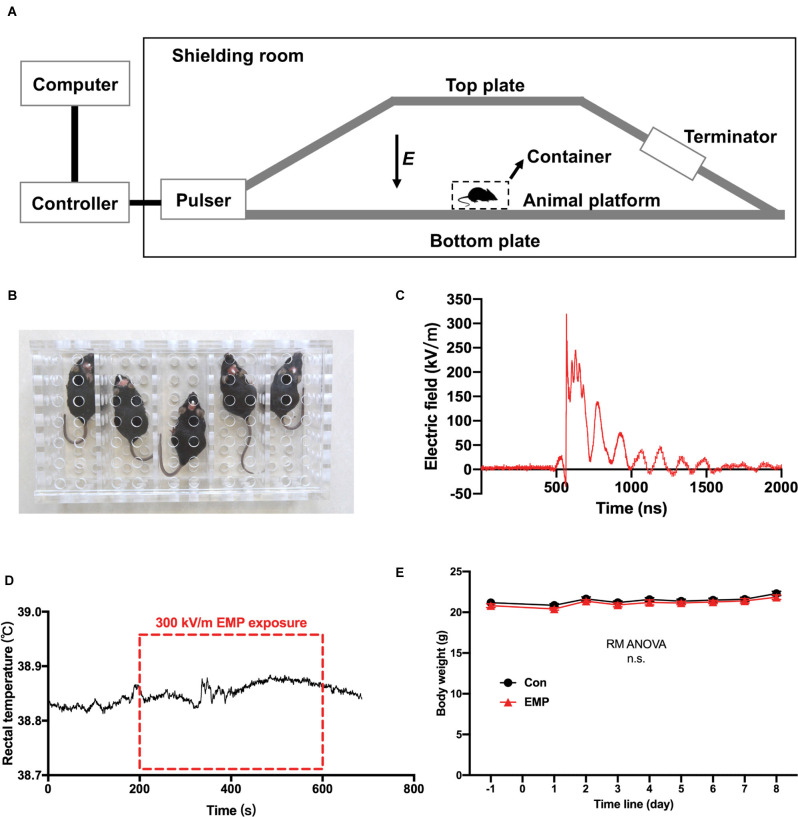
The electromagnetic pulse (EMP) exposure settings and the effects of EMP on the rectal temperature and body weight in mice. **(A)** Schematic diagram of EMP simulator. **(B)** Mouse container, a square metal-free box made of resin. **(C)** Measured electric field waveform. **(D)** Rectal temperature of mice before, during, and after EMP exposure (*n* = 1 mouse). **(E)** Body weight (*n* = 12 mice per group), presented as mean ± SEM. Repeated-measures analysis of variance (RM ANOVA) was used to evaluate the difference between the two curves in **(E)**. n.s., not significant.

The EMP generator was established by the Beijing Institute of Radiation Medicine, based on the international standards described in IEC 61000-2-9 (Figure 2A). When exposed to EMPs, mice were fixed in a square metal-free box (Figure 2B), and placed on the animal platform of the EMP generator where the two plates were parallel and the electric field between them was uniform. The mice in the EMP-exposed group received whole-body exposure to 400 times of EMPs with the same setting at a repetition frequency of 1 Hz. The center frequency of the EMP was 100 MHz. The measured parameters of single EMP waveform were as follows: the rising edge is 2.8 ns, and the amplitude peak value was approximately 320 kV/m, as shown in Figure 2C. The mice of the control were processed in parallel with those in the EMP-exposed group, but with the EMP generator switched off. It was found that the increase of rectal temperature in mice induced by 300 kV/m EMP radiation was lower than 0.1°C by a fiber-optic thermometry (model THR-NC-1084C, FISO, CA), as presented in Figure 2D. A general weight gain over the course of 8 days after radiation for both cohorts was observed, which was attributed to the physiological conditioning. There was no significant difference in the body weight between the EMP-exposed and the control groups (Figure 2E).

### Morris Water Maze (MWM)

The maze was an open circular pool with a diameter of 100 cm filled halfway with water, and was equally divided into four quadrants (I, II, III, IV). The water temperature was controlled at 20°C ± 2°C. The escape platform had a diameter of 6 cm and was submerged 1 cm below the water surface. A monitoring camera dedicated to animal behaviors was installed on the top of the maze, and the Anymaze software (Version 6.32, Stoelting, USA) was used to record the motion trajectory of animals.

Training were carried out before EMP exposure, once a day for 3 days. Mouse was put into the pool from each of the four quadrants. If the mouse found the platform within 60 s, then continued to stay on the platform for another 15 s. If not, the mice were guided to the platform to stay for 15 s. The interval between two tests of each mouse was more than 20 min to restore the animal’s physical strength. The main purpose of the 3-day training before EMP radiation was to habituate the mice to water and handling to reduce the stress during the formal tests. And the mice with poor swimming ability and lack of cooperation were excluded during this process. Moreover, the learning and memory ability in mice was evaluated to eliminate the influence of individual differences between groups on test results.

From day 1 to day 4 post radiation, the platform was placed in the quadrant I and hidden platform test was conducted. The mice were put into the pool from each of the four quadrants, and the starting quadrant was determined randomly. The time taken by the mice to find the platform was recorded within 60 s. If the mice did not find the platform within 60 s, the escape latency was recorded as 60 s. On day five post exposure, the platform was removed and probe trials were performed. The mice were put into the pool from the contralateral quadrant (III), and were allowed to explore freely in the pool for 60 s.

Next, a reversal spatial learning test, which allows assessing the cognitive flexibility necessary to extinguish old memories and form a new memory, was carried out (Vorhees and Williams, 2006; Carvalho da Silva et al., 2022). The platform was moved to the quadrant III, the hidden platform experiment was conducted from day 6 to day 8 post exposure, and the probe test was carried out on day 9 post exposure. The methods were the same as before.

### Y Maze, Novel Arm Discrimination

Spatial reference memory, which is underlined by the hippocampus, can be tested by novel arm discrimination test (Kraeuter et al., 2019b). A Y-maze was divided into the start arm, the novel arm, and another arm, with different spatial markers located inside. The angle of each arm was 120 degrees, and the size of each arm was 30 cm long × 8 cm wide × 15 cm high. The mice were first placed in the behavioral room for 30 min every day for three consecutive days to reduce the fear levels of the animals. The Y-maze test consisted of two stages. In the first stage, a baffle was used to close the novel arm, and the mouse was placed into the Y-maze from the start arm and was allowed to freely explore in the start arm and another arm for 5 min. One hour later, the second stage was initiated. The novel arm was opened, and the mouse was placed in the start arm again and allowed to freely move for 5 min. Before each test with a new animal, the behavioral box was wiped with alcohol to eliminate the effect of odor. ANY-maze software was used to record the locomotor tracks of the mice.

### Novel Object Exploration (NOE)

In order to assess the novelty-seeking behavior in mice, a novel object exploration test was carried out, see also Río-Alamos et al. (2015) and Cuenya et al. (2016). In the test phase, the mice were first placed in a 40 cm × 40 cm square opening to move freely for 5 min, after which a novel 4 cm × 4 cm × 4 cm cube was placed in the center of the opening, and the mice were allowed to continue to freely explore for 5 min. Before each test with a new animal, the behavioral box was wiped with alcohol to eliminate the effect of odor. ANY-maze software was used to record and analyze the number of entries and the time periods that the mice contacted the novel object.

### Shuttle Box

The shuttle box consisted of two compartments of the same size on the left and right sides (20.3 cm × 15.9 cm × 21.3 cm). The two compartments were connected by an arched door. One side of the compartment was connected to the stimulator, which can deliver electric shocks to the feet of the mice and was considered the electric shock zone; the other side of the compartment had no electric shocks and was the safe zone. Before the experiment, the animals were placed in the test box to move freely for 5 min to eliminate the exploratory reflex. In the experimental test, the mice were first placed in the electric shock area of the shuttle box, and the conditioned buzzer sound was initially provided for 5 s, followed by electric stimulation for the next 10 s. If the mouse escaped to the safe area within 5 s of the sound stimulation, it was considered an active escape response. If the mouse escaped to the safe area after the electric shock, it was considered a passive escape response. Tests were performed from 1 day to 14 days after EMP exposure, and 50 trains were performed per day each animal.

### Open Field (OF)

The OF box consisted of a 40 cm long × 40 cm wide × 35 cm high square box. The center 20 cm × 20 cm was defined as the central area, and the remaining was considered the surrounding area. Environmental adaptation was performed before the start of the experiment, and the method was the same as was previously described (Kraeuter et al., 2019a). During the test, the mice were placed in the center area, and were allowed to freely explore for 5 min. ANY-maze software was used to record and analyze the movement of the mice.

### Elevated Plus Maze

The elevated plus maze consisted of two open arms and two closed arms, which were perpendicular to each other and formed a cross. The arm was 65 cm long × 5 cm wide. There is no shielding around the open arm, and the shielding around the closed arm is 15 cm high. The height of the maze was 55 cm above the ground. Environmental adaptation was performed before the start of the experiment, and the method was the same as was previously described (Walf and Frye, 2007). During the test, the mouse was placed in the central area of the maze with the head facing the open arm. The mouse was allowed to move freely in the maze for 5 min. ANY-maze software was used to track the movement trajectory of the mice.

### Virus Injection and Optical Fiber Implantation

The virus AAV-Syn-jGCaMP7f-WPRE used in the study was provided by OBiO Technology (Shanghai) Corp., Ltd. (Shanghai, China). Namely, GCaMP7f was cloned into the AAV serotype2/9-Syn vectors, with the final titers up to 6.54 × 10^12^ viral particles/ml. Meanwhile, AAV vectors carrying Syn-EGFP (OBiO Technology) were applied, and GFP-expressing animals were used as controls for comparison with GCaMP7f-expressing animals (Li et al., 2019; Supplementary Figure 1).

Virus injection and fiber implantation was performed as described previously (Liu et al., 2014; Zhang et al., 2016; Wei et al., 2018). Mice were anesthetized with approximately 1% isoflurane inhalation (RWD Life Science, Shenzhen, China), after which the head of the mouse was mounted onto a stereotaxic instrument (RWD Life Science) on a heat pad. Each eye of the animal was coated with erythromycin eye ointment for protection. The scalp was shaved and then incised through the midline. A small hole on the skull was bored gently with a mini drill (Strong204, Saeshin, South Korea). AAV vectors (200 nl) were slowly infused through a sharp glass pipette into the hippocampal CA1 region of the mouse [-2.70 mm anteroposterior (AP), 2.4 mm mediolateral (ML), -1.70 mm dorsoventral (DV) relative to the bregma] at rate of 10 nl·min^−1^, by a microsyringe pump (KDS LEGATO 130, RWD Life Science). After the infusion was completed, the glass pipette remained still at the injection site for another 10 min and then were slowly withdrawn. Immediately following virus infusion, an optical fiber (200 μm outer diameter, 0.37 numerical aperture) bounded to a ceramic ferrule was implanted with its tip targeting at the virus injection site. The ceramic ferrule was secured to the skull using dental acrylic. After the viruses were stably expressed (approximately 2 weeks later), further tests were conducted.

### Fiber Photometry

The fiber photometry of GCaMP signals was conducted by a multichannel fiber photometry system (Thinker Tech Nanjing Bioscience Inc, Nanjing, China), as described previously (Liu et al., 2018; Luo et al., 2018; Dong et al., 2020). Briefly, an optical fiber (220 μm outer diameter, 0.37 numerical aperture) was used to transmit light between the fiber photometry system and the implanted optical fiber in mice. A laser beam of 480 nm produced by the semiconductor laser was reflected by a dichroic mirror and transmitted to excite GCaMPs expressed in the brain of mice. The optical power at the tip of the fiber was set to 30 μW to minimize bleaching. Fluorescence generated after excitation of GCaMP was collected by the optical fiber, passed through the dichroic mirror, then detected by the sensor of a CMOS camera of the fiber photometry system. A Lab view program (Thinker Tech Nanjing Bioscience Inc, Nanjing, China) was developed to control the CMOS camera and record calcium signal at a frequency of 50 Hz.

The effects of real-time 300 kV/m EMP exposure on the calcium activity of hippocampal CA1 neurons were detected by the fiber photometry system. The method was conducted in the following manner. First, mice were trained to adapt to the optical fiber connected to the head. Thereafter, the mouse was placed in a plastic open field box (40 cm × 40 cm, free of metals). The open field box was placed on the EMP exposure platform, and the mouse was able to move freely in the open field. GCaMP signals were recorded 5 min before, during, and 5 min after EMP exposure.

Besides, the experiments were performed before radiation and on day 1 and day 7 after irradiation. The mouse was connected to the fiber photometry system and placed in an open field box, and fluorescence signals were continuously collected for 5 min. Then, a 4 cm × 4 cm × 4 cm cube was placed in the center of the open field box as a novel environmental stimulus, the recording was continued for another 5 min. The behavior event signal was recorded synchronously.

### Frozen Section and GCaMP Observation

After the completion of fiber photometry testing, mice were deeply anesthetized with 0.5% pentobarbital sodium (i.p., 80 mg/kg) and decapitated. The brains were harvested, and coronally sectioned into 30 μm slices by a cryostat (CM1950, Leica). After being stained with DAPI, the expression of GCaMP was observed under a microscope (Leica).

### Hematoxylin and Eosin (H&E) Staining

Mice were anesthetized with 0.5% pentobarbital sodium (i.p., 80 mg/kg) and sacrificed *via* decapitation. Brain tissues were isolated on ice. Right hemispheres of mice were fixed in 10% buffered formalin, dehydrated, and embedded. Paraffin sections were prepared with a thickness of 3 μm, stained with H&E, and photographed by using an optical microscope (Leica).

### Transmission Electron Microscopy

Mice were anesthetized with 0.5% pentobarbital sodium (i.p., 80 mg/kg) and sacrificed *via* decapitation. Brain tissues were isolated on ice, and tissue blocks with sizes of 1 mm^3^ were taken from the dorsal hippocampus of the left hemisphere. After being fixed with 2.5% glutaraldehyde and post-fixed with 1% osmium acid, tissues were dehydrated with an ethanol gradient, embedded, and cured to produce ultrathin sections. The sections were then double-stained with uranyl acetate and lead citrate, after which they were observed and photographed using a transmission electron microscope (Hitachi, Japan).

### Fiber Photometry Analysis and Statistical Tests

The data produced by fiber photometry were processed as previously described (Li et al., 2016; Zhong et al., 2017; Dong et al., 2020). The changes in the GCaMP7f fluorescence intensity (△F/F_0_) were quantitatively analyzed by using MATLAB 2016a (MathWorks, Cambridge, United Kingdom). △F/F_0_ = (V_signal_ - F_0_)/F_0_, where F_0_ was the baseline fluorescence signal averaged over a 2.0-s-long control time window and V_signal_ was the peak value of the fluorescence signal. And the area under the curve (AUC) between the starting point of the fluorescence signal (0 s) and subsequent 10 s was calculated to verify the strength of activation or inhibition of calcium signal in neurons.

All data in this study are presented with Mean ± Standard Errors of Mean (SEM). Student’s *t*-test was used to compare the two groups. Repeated-measures analysis of variance (RM ANOVA) was performed to analyze repeated measurement data. One-way analysis of variance (one-way ANOVA) followed by Bonferroni’s *post-hoc* tests was used to compare multiple group data. Student’s *t*-test was performed to compare the differences between two groups. The statistical analysis was conducted using SPSS software (IBM, Armonk, NY, USA). Statistical differences were considered when *p* < 0.05.

## Results

### EMPs Impaired the Spatial Learning and Memory Ability in Mice

**Figure 3:**
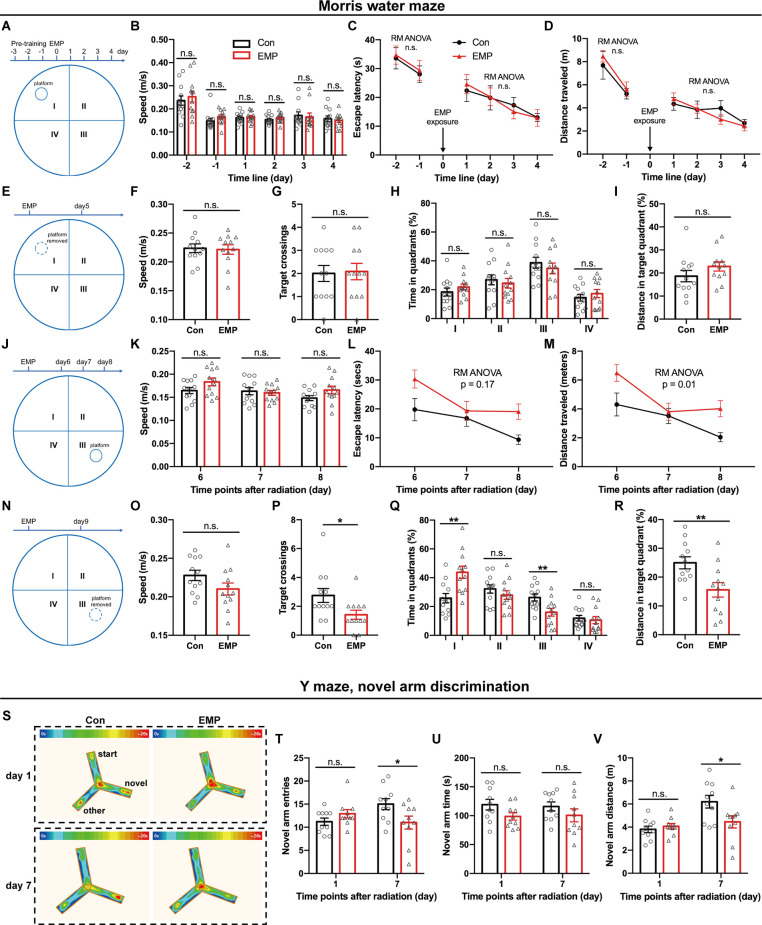
EMPs impair the spatial learning and memory ability in mice. **(A–R)** Morris water maze (MWM) experiment (*n* = 12 mice per group). From day 1 to day 4 post exposure, the platform was placed in the quadrant I and hidden platform tests were conducted, and the speed, escape latency, and distance traveled in locating the platform were analyzed **(A–D)**. On day 5 post exposure, the platform was removed and probe tests were performed, and the speed, target crossings, time (%) in each quadrant, and distance (%) in the target quadrant (I) were shown **(E–I)**. From day 6 to day 8 post exposure, the platform was moved to the contralateral quadrant (III), and hidden platform tests were conducted, the speed, escape latency, and distance traveled in locating the platform were presented **(J–M)**. On day 9 post exposure, the platform was removed, the probe tests were conducted, and the speed, target crossings, time (%) in each quadrant, and distance (%) in the target quadrant (III) were calculated **(N–R)**. **(S–V)** Y maze, novel arm discrimination (*n* = 10 mice per group). The average heat map of movement was calculated (S), and the novel arm entries, time and distance were analyzed **(T–V)**. Con, the control group; EMP, the 300 kV/m EMP-exposed group. All data are presented as mean ± SEM. Student’s *t*-test was used to compare the two groups **(B–V)**. Repeated-measures analysis of variance (RM ANOVA) was used to evaluate the difference between the two curves in **(C,D)** and **(L,M)**. *p < 0.05; **p < 0.01; n.s., not significant.

The brain is considered to be sensitive to EMR, as many studies have reported the damaging effects of EMR on the brain (Shahin et al., 2018; Narayanan et al., 2019; Gökçek-Saraç et al., 2021; Hu et al., 2021; Tan et al., 2021). Learning and memory are the basis of brain cognitive function. Morris water maze is the preferred classical experiment for studying the spatial learning and memory ability of mice. In this study, the platform was first placed in the quadrant I, and pre-training was performed from day 3 to day 1 before EMP radiation, and the hidden platform tests were conducted from day 1 to day 4 after EMP exposure. There was no difference between two groups in the swimming speed, average escape latency, and distance traveled during the pre-training phase on day 1 and day 2 before exposure (Figures 3A–D). There were no obvious changes observed in the swimming speed of EMP-irradiated mice, thus indicating that EMP exposure did not have an effect on the motor function of mice. The average escape latency and the distance traveled in locating the platform showed no significant differences between the EMP-exposed group and the control group (Figures 3A–D). On day 5 after radiation, the platform was removed for the probe tests. The target crossings, the time (%) and distance (%) in the target quadrant (I) did not change significantly in mice exposed to EMPs (Figures 3E–I). Afterwards, the platform was moved to the contralateral quadrant (III), the performance of the mice in the EMP group was markedly worse than that in the control group. In the hidden platform tests performed on day 6, 7, and 8 after radiation, the average escape latency in mice of the EMP group was extended, and the distance traveled was increased (Figures 3J–M). In the probe tests conducted on day 9 after radiation, mice of the EMP group traversed the platform less frequently, the time (%) and distance (%) spent in the target quadrant (III) were decreased, in contrast the time (%) spent in the contralateral quadrant (I) was increased (Figures 3N–R). There was evidence the preference for the target annulus was highest during the first 30 s of the probe trial, and was lower during the next 30 s interval (Blokland et al., 2004), which might explain the low preference of mice in the target during the 60-s probe tests presented in Figures 3H–Q. The above results indicated that 300 kV/m EMPs could cause a reduction in the spatial learning and memory ability in mice.

The Y-maze novel arm experiment utilizes the habit of rodents to explore new environments, and is usually used to evaluate the spatial learning and memory ability in rat and mouse. Compared with that of the Con group, the number of times to enter the novel arm, and the time and distance spent in the novel arm showed no difference in mice of the EMP group. However, the novel arm entries and distance traveled were significantly reduced at 7 days in mice exposed to EMPs (Figures 3S–V). These results indicated that the spatial recognition and memory ability of mice were reduced after 300 kV/m EMP irradiation, which was consistent with the that of the Morris water maze experiment.

### EMPs Showed on Effects on the Avoidance Learning, Novelty-Seeking and Anxiety Behaviors in Mice

**Figure 4:**
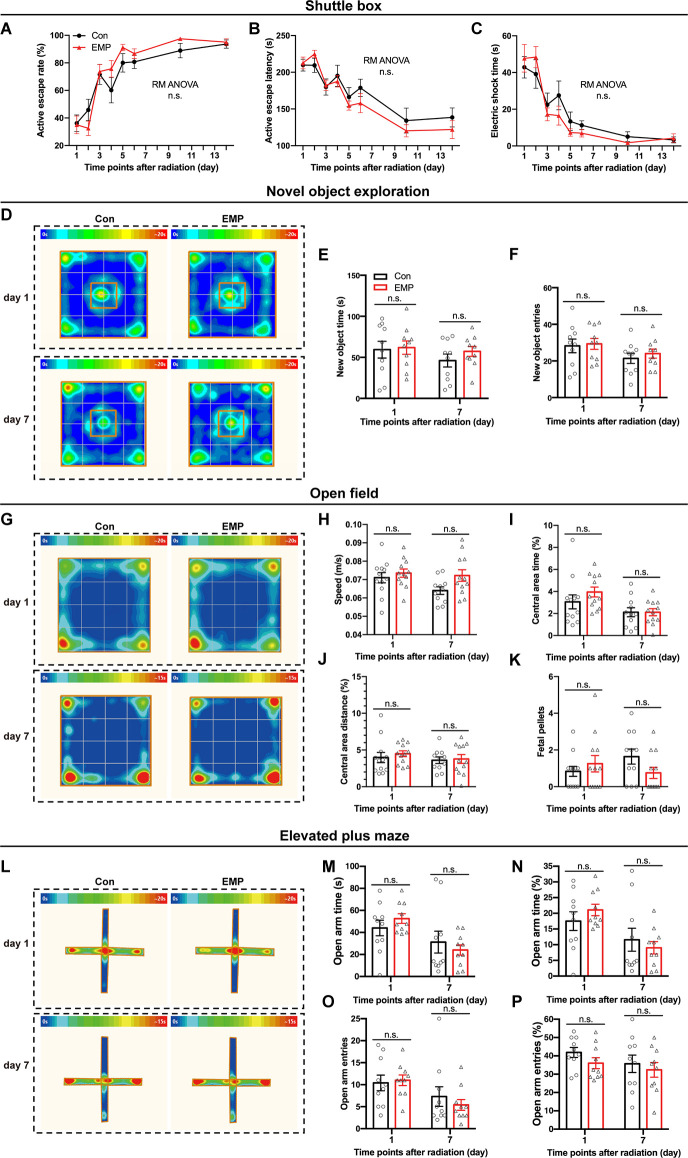
EMPs show no significant effects on the avoidance learning, working memory and anxiety state in mice. **(A–C)** Shuttle box (*n* = 12 mice per group), active escape rate **(A)**, active escape latency **(B)**, electric shock time **(C)**. **(D–F)** New object exploration (*n* = 10 mice per group), average heat map of motion trajectory **(D)**, new object time **(E)**, new object entries **(F)**. **(G–K)** Open field experiment (*n* = 12 mice per group), average heat map of motion trajectory **(G)**, speed **(H)**, central area time % **(I)**, central area distance % **(J)**, and fetal pellets **(K)**. **(L–P)** Elevated plus maze (*n* = 10 mice per group), average heat map of the motion trajectory **(L)**, open arm time **(M)**, open arm time % **(N)**, open arm entries **(O)**, and open arm entries % **(P)**. All data are presented as mean ± SEM. Repeated-measures analysis of variance (RM ANOVA) was used to evaluate the difference between the two curves in **(A–C)**. Student’s *t*-test was used to compare the two groups **(E–P)**. n.s., not significant.

The shuttle box allows for animals to avoid harmful stimuli through learning, which is an important means for quantitatively measuring learning ability of classical conditioned reflex. In this study, there were no significant differences in the active escape rate, active escape latency, and electric shock time between the two groups treated with EMP radiation or not (Figures 4A–C). These results indicated that 300 kV/m EMPs had no significant effect on the avoidance learning ability of the classical conditioned reflex in mice.

The novel object exploration experiment is a test based on the principle that animals have an innate tendency to explore novel environmental stimuli, which reflects the novelty-seeking behavior in mice. Compared with that of the control, there were no significant differences in the time and frequency of contacting the novel object in the EMP-exposed mice (Figures 4D–F). These results indicated that EMP radiation showed no significant effects on the novelty-seeking behavior in mice.

The open field experiment utilizes the psychological conflict that the animals were afraid of the open environment but liked to explore, which can assess changes in the animal’s anxiety behavior. No significant changes occurred in the average speed, central area time and distance, and fetal pellets in mice exposed to EMPs (Figures 4G–K). These results indicated that 300 kV/m EMP exposure did not cause anxiety-like behaviors in mice.

Same as the open field test, the elevated plus maze can investigate the animal’s anxiety state in which the animal’s exploration characteristics of novel environments and the fear of hanging open arms form conflicting behaviors. After EMP radiation, no significant difference was observed in the open arm time (%) and entries (%) of mice (Figures 4L–P). The elevated plus maze test results showed that 300 kV/m EMPs did not cause anxiety-like behaviors in mice, which was consistent with the results obtained by the open field experiment.

### Real-Time 300 kV/m EMP Exposure Disturbed Neuronal Activities in Mouse Hippocampal CA1

**Figure 5:**
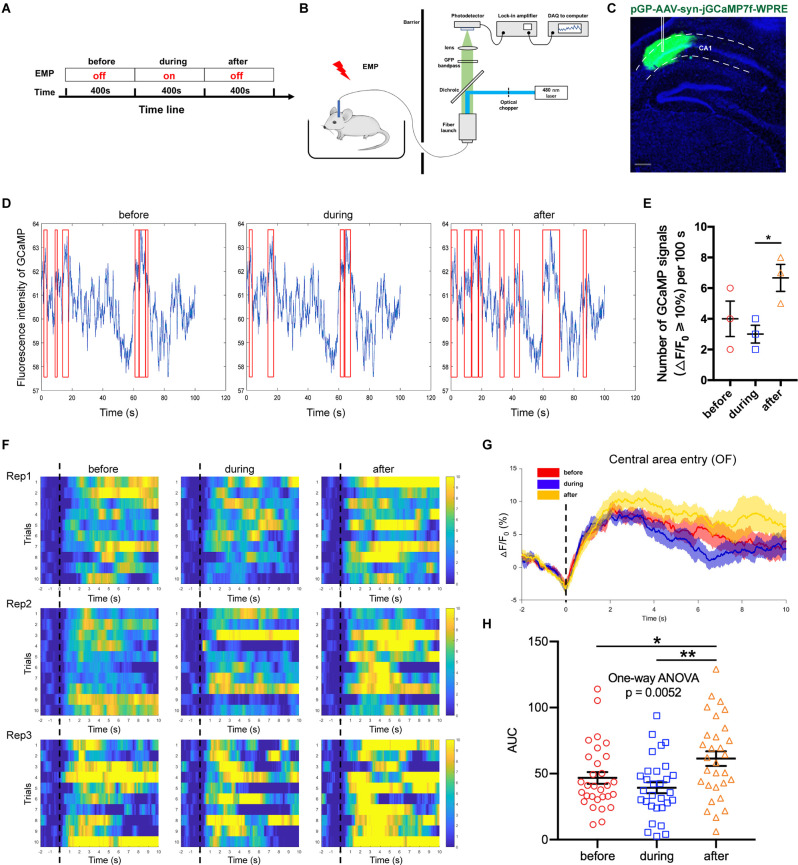
EMP real-time exposure disturbed neuronal activities in hippocampus CA1 (*n* = 3 mice). **(A)** Timeline of the experiment, “before” indicates 400 s prior to EMP exposure, “during” indicates 400 s during real-time EMP exposure, “after” indicates 400 s after EMP radiation end. **(B)** Schematic of the experimental setup for fiber photometry of neuronal GCaMP fluorescence from freely behaving mice in an open field (OF) receiving EMP exposure. **(C)** Representative photos of GCaMP *in situ*, showing that the virus AAV-syn-jGCaMP7f-WPRE was well expressed in the CA1 area of dorsal hippocampus, with accurate localization and no diffusion. **(D)** Representative images of the measured fluorescence intensity of GCaMP in hippocampal CA1 before, during and after EMP exposure. Red box indicates the neuronal calcium signals with amplitude ≥ 10% (offset = 20). **(E)** Statistical analysis of the number of GCaMP signals with △F/F_0_ ≥ 10% per 100 s before, during and after EMP exposure, as presented in **(D)**, from three biological replicates. **(F)** Heat map of calcium amplitude △F/F_0_ (%) of hippocampal CA1 neurons from three biological replicates before, during and after EMP exposure. **(G)** Plot of average calcium amplitude △F/F_0_ (%) of three biological replicates before, during and after EMP exposure. **(H)** Plot of area under the curve (AUC) of neuronal calcium signals from multiple trials on three biological replicates before, during, and after EMP exposure. 0 s for △F/F_0_, marked by dotted lines, indicated that mice were entering the central area of OF. The data in **(E–H)** are presented as mean ± SEM. One-way ANOVA followed by Bonferroni’s *post-hoc* tests were used to compare the differences between multiple groups in **(E–H)**. *p < 0.05; **p < 0.01.

Adeno associated virus (AAV) vectors can be used to express the calcium-sensitive fluorescent protein GCaMP in neurons, the fluorescence intensity of which is positively correlated with the concentration of cytoplasmic calcium ions. Fiber photometry is utilized to record the changes in the fluorescence intensity, thus enabling the detection of *in vivo* neuronal activities. This method owns high temporal and spatial resolution; additionally, it is compatible with the electromagnetic environment. In this study, AAV-Syn-jGCaMP7f-WPRE was used to infect neurons in the CA1 region of the dorsal hippocampus. The promoter used was the neuron-specific promoter synapsin (Syn), which could express GCaMP in all types of neurons. On this basis, this study used the fiber photometry system to observe the effect of 300 kV/m EMP real-time exposure on the neuronal calcium activity of hippocampal CA1 (Figures 5A–C). We found that the activation frequency of hippocampal CA1 neurons decreased during real-time radiation, but increased in a short time after the end of radiation (Figures 5D,E). Through the analysis of the calcium amplitude △F/F_0_ (%) in multiple trials, it was found that 300 kV/m EMP real-time exposure seemed to inhibited the strength of neuronal calcium activities in the hippocampal CA1 area. However, immediately after radiation, the calcium amplitude of the hippocampal CA1 neurons was significantly increased (Figure 5F–H). These results indicated that real-time 300 kV/m EMP exposure could lead to the disorder in neuronal activities of hippocampal CA1, which was inhibited during EMP radiation but activated immediately after exposure.

### Neuronal Activities in Hippocampal CA1 Were Inhibited After 300 kV/m EMP Radiation

**Figure 6:**
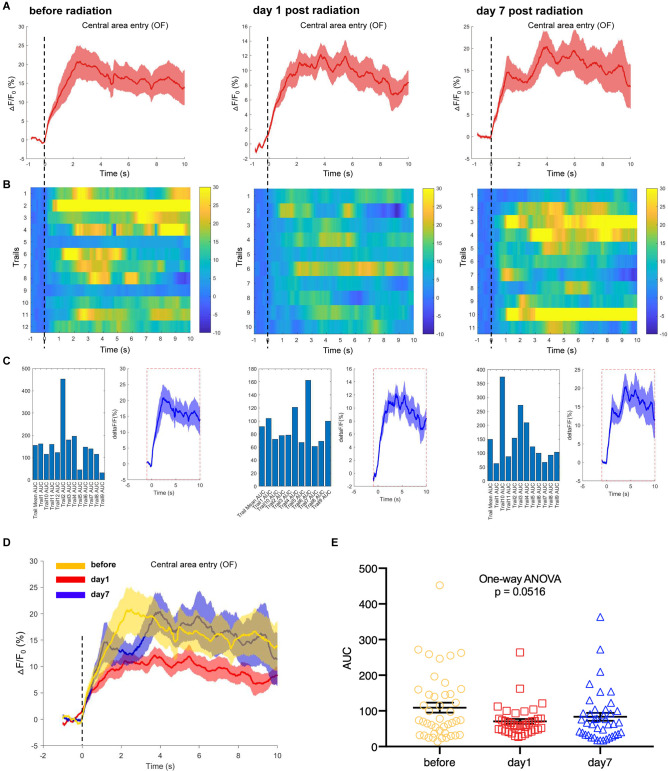
EMP exposure inhibits neuron activities in the hippocampal CA1 area when mice entered the central area in an open field (OF, *n* = 3 mice). **(A)** Plot of calcium amplitude △F/F_0_ (%) of hippocampal CA1 area, before radiation and on day 1 and day 7 after radiation. The red line is the mean of different trials from a single mouse, and the shaded part represents the standard error. **(B)** Color-coded intensity of calcium transients from different trials, corresponding to each signal presented in **(A)**. **(C)** The area under the curve (AUC) between cue onset (0 s) and subsequent 10 s from different trials as shown in **(B)**. **(D)** The combined line diagram of calcium amplitude △F/F_0_ (%) in CA1 neurons as shown in **(A)**. **(E)** Plot of AUC in multiple trials from three biological replicates before and different time points after exposure. 0 s for △F/F_0_, marked by dotted lines, indicated that mice were entering the central area of OF. The data in **(A–E)** are presented as mean ± SEM. One-way ANOVA followed by Bonferroni’s *post-hoc* tests were performed to evaluate the differences between multiple groups in **(E)**.

Additionally, we used the fiber photometry technique to track the changes in the neuronal calcium activities of hippocampal CA1 area at different time points after EMP exposure. First, the mice were placed in an open field box, and the neuronal calcium activity of the hippocampal CA1 was collected at the corresponding time points before, day 1 and day 7 after EMP radiation. The average of △F/F_0_ of neuron calcium activities of CA1 on day 1 after EMP exposure was lower than that prior to exposure, as shown in Figures 6A–D. Additionally, the AUC of multiple trials from three biological replicates decreased on day 1 after radiation, and a slight recovery occurred on day 7, which slightly missed the margin of significance (One-way ANOVA, *p* = 0.0516, Figure 6E). We therefore concluded that EMP radiation could inhibit the neuron activities in CA1 in the open field test, especially on day 1 after EMP exposure.

**Figure 7:**
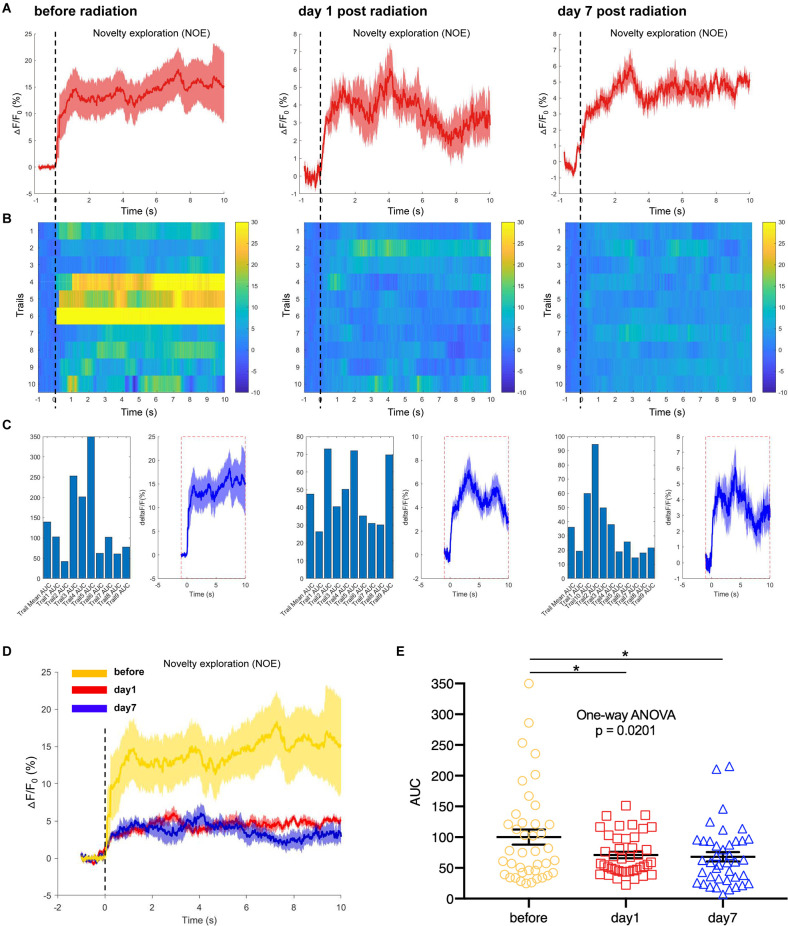
EMP radiation inhibits neuron activities in the hippocampal CA1 area when mice were contacting a novelty during novel object exploration (NOE, *n* = 3 mice). **(A)** Plot of calcium amplitude △F/F_0_ (%) of hippocampal CA1 area, before radiation and on day 1 and day 7 after radiation. The red line is the mean of different trials from a single mouse, and the shaded part represents the standard error. **(B)** Color-coded intensity of calcium transients from different trials, corresponding to each signal presented in **(A)**. **(C)** The area under the curve (AUC) between cue onset (0 s) and subsequent 10 s from different trials as shown in **(B)**. **(D)** The combined line diagram of calcium amplitude △F/F_0_ (%) in CA1 neurons as shown in **(A)**. **(E)** Plot of AUC in multiple trials from three biological replicates before and different time points after EMP exposure. 0 s for △F/F_0_, marked by dotted lines, indicated that mice were exploring a novelty (NOE). The data in **(A–E)** are presented as mean ± SEM. One-way ANOVA followed by Bonferroni’s *post-hoc* tests were performed to evaluate the differences between multiple groups in **(E)**. *p < 0.05.

In another test, a novel object was selected as a novel environmental cue and put into the open field, to detect the responses of hippocampal CA1 neurons to the novelty in mice. We recorded and analyzed the neuronal calcium activities in hippocampal CA1 area when mice were completing the task of novel object exploration. The results showed that the CA1 neuronal calcium activities when mice were contacting the novel object were significantly inhibited on day 1 and day 7 after EMP irradiation (Figures 7A–E). The hippocampus is required for episodic memory, and hippocampal CA1 neurons are reported to shown place cell activity and sensitive to spatial cues (Lisman et al., 2017; Rolls and Wirth, 2018). The inhibition of calcium activity in CA1 neurons after EMP radiation when completing novel object exploration task indicated that the responses of CA1 neurons to spatial novel stimuli were weakened after EMP radiation, which might be an important reason explaining the impaired spatial learning and memory ability in mice exposed to EMPs.

### EMPs Damaged Neuron Structures in the Hippocampal CA1 Region of Mice

**Figure 8:**
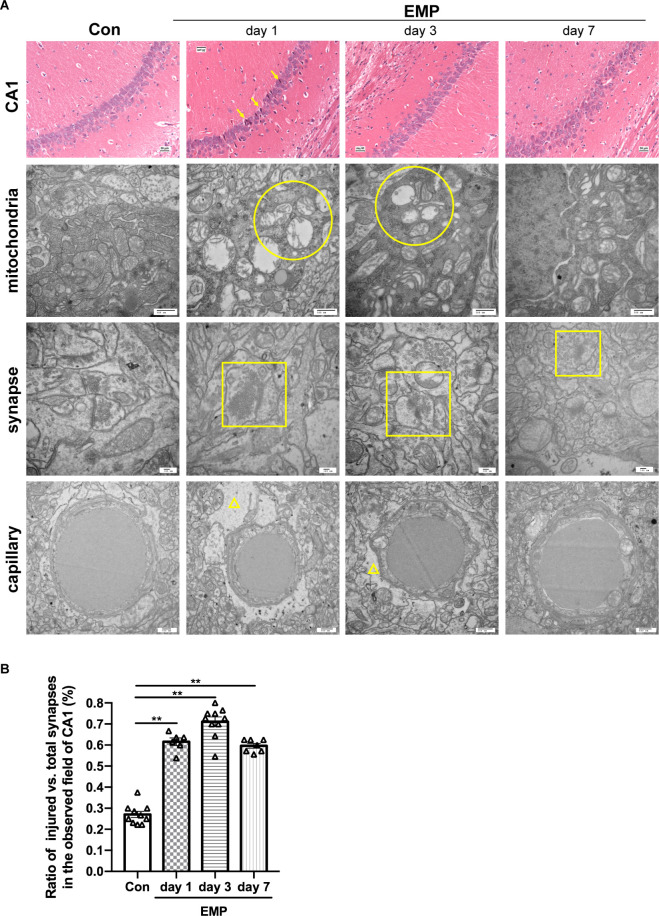
EMP exposure damages the structures of hippocampal CA1 neurons in mice (*n* = 3 mice per group). **(A)** On day 1, day 3, and day 7, mice were anesthetized with pentobarbital sodium and then decapitated. The brain was isolated and stained with H&E. The structures of the hippocampi were observed under an optical microscope, and representative images of the CA1 area were presented. The ultrastructure of CA1 neurons was observed by transmission electron microscope, and representative images of the mitochondria, synapses, and capillary were shown. Scale bar = 30 μm for H&E, 500 nm for transmission electron microscopy. Arrows, the damaged neurons in CA1; circular, the mitochondria with swelling, crista breakage, and fragmentation; square, the injured synapses; triangle, the widened perivascular space. **(B)** Statistical analysis of the ratio of injured vs. total synapses in hippocampus of different groups. Two to four fields of hippocampal CA1 per mice from three biological replicates was used for quantitative analysis. The data are presented as mean ± SEM. Student’s *t*-test was performed to evaluate the differences between the EMP-exposed groups at different time points with the Con. **p < 0.01.

The hippocampal CA1 area of the mice in the control group showed a normal morphological structure, and the main neuron type included pyramidal neurons, with large, round, lightly stained nuclei and weakly eosinophilic cytoplasm. In the EMP group, the hippocampal CA1 area showed obvious injury changes on day 1 post exposure, characterized by cell body sizes of neurons, eosinophilic staining of the cytoplasm, and pyknosis and hyperchromatic nuclei, which exhibited triangular or fusiform shapes. A recovery trend was observed on day 3 and day 7 after radiation. Through transmission electron microscopy, impaired mitochondria were observed on day 1 and day 3 after radiation, mainly manifested as swelling, crista breakage, and fragmentation in morphology, indicating the abnormal mitochondria functions and energy metabolism disorder in neurons induced by EMPs. The synaptic gaps of neurons exposed to EMPs were blurred, and the synaptic vesicles seemed to accumulated, which could result in the disturbed synaptic transmission at the level of neural circuit. Moreover, we found that the ratio of the damaged synapses in hippocampus was significantly increased from day 1 to day 7 after radiation (Figures 8A,B). Besides, the perivascular space in the hippocampus was widened, which might be attributed to the increased vascular permeability and the destructive blood-brain barrier caused by EMP radiation, as previously reported (Ding et al., 2010; Qiu et al., 2010; Zhou et al., 2013, 2017; Li et al., 2018; Gao et al., 2020). On the whole, the above changes were more obvious on day 1 and day 3 after radiation, and showed a decreasing trend on day 7 after radiation (Figure 8). Additionally, the damaged hippocampal structures showed a recovery trend on day 7 pyknosis, however the hippocampal neuronal activities were still inhibited at that time, which could be explained by the unrecovered neural circuit function, especially the unrecovered synaptic connection as presented in Figure 8B.

## Discussion

Generally, the toxic effects of EMR are classified as thermal and non-thermal effects, based on whether the observed effect was a result of a significant temperature change (thermal effects) or independent of any change in temperature considered in excess of thermal noise (nonthermal effects; National Toxicology Program). EMPs are high-energy, nonionizing radiation that can generate a strong instantaneous electromagnetic field and have a wide spectrum. Due to the extremely low duty cycle, the body absorbs less energy in total and hardly change in temperature in response to 300 kV/m EMPs, as proved in this study. Therefore, the biological effects of EMPs are mainly attributed to the non-thermal effects.

The brain is recognized as being one of the target organs that are sensitive to EMR. Numerous studies have shown that EMR can cause cognitive impairment in the brain under certain conditions (Shahin et al., 2018; Narayanan et al., 2019; Gökçek-Saraç et al., 2021; Hu et al., 2021; Tan et al., 2021). There are few studies regarding the effects of EMPs on behavior and nerves, and further investigations are required. Jiang et al. used 50 kV/m, 100 Hz EMPs to irradiate Sprague Dawley (SD) rats and found that long-term exposure to the EMP environment could cause impairment of spatial learning and memory in rats and the inhibition of autonomous exploration behavior (Jiang et al., 2016). In this study, C57BL/6N male mice were irradiated with EMPs with peak power of 300 kV/m, and the effects of EMPs on spatial learning and memory ability, novelty-seeking behavior, active escape responses, and anxiety-like behaviors were studied. It was found that EMPs could cause a decrease in the spatial learning and memory ability in mice, especially when mice performed difficult tasks, suggesting that EMPs under this condition caused a reduction in the redundancy of the nervous system function in mice. Besides, EMPs had no significant effects on the novelty-seeking behavior, avoidance learning and memory ability of mice. We speculated that the damages of EMPs to the nervous system were slight or within the range of redundancy, and there were no obvious damage phenotypes when the mice were dealing with some simple tasks. In addition, after EMP exposure, the mice did not demonstrate an obvious anxiety state, so that the effect of EMPs on the spatial learnng and memory ability of mice was not disturbed by anxiety.

At neuron levels *in vivo*, our study reported the evidence that EMPs caused disturbed neuronal calcium activities in different patterns under cases of real-time exposure to EMPs and post-exposure. Spatial learning and memory are critical to the formation of navigation and situational memory. The synaptic structural and functional plasticity of hippocampal neurons is the neurobiological basis for spatial representation and navigation (Lisman et al., 2017; Rolls and Wirth, 2018). Among them, the hippocampal CA1 area is an important brain area responsible for spatial learning and memory, where the pyramidal neurons are place cells that response to spatial cues. The inhibition of the pyramidal neurons in the hippocampal CA1 area through electrophysiological, genetic, or drug factors can lead to spatial learning and memory dysfunction (Moser et al., 2015; Cobar et al., 2017). Many studies have shown that a good correspondence exists between calcium activities and neuroelectric signals in most nerve cells, founding the basis of behaviors at the cell level (Fosque et al., 2015; Lagache et al., 2021). Through fiber photometry technique, we first found that real-time exposure of 300 kV/m EMPs caused abnormal neuronal calcium activities in CA1 area, which was inhibited during EMP radiation but markedly activated immediately after radiation end. Consequently, the overactivation of pyramidal cells subsequent to EMP exposure, the most important neurons in the hippocampal CA1 area, could result in a large release of glutamate, which in turn caused excitotoxicity and neuronal damages (Sanches et al., 2021; Toro-Fernández et al., 2021). There is evidence that pulsed electromagnetic field can change the potential on the plasma membrane by affecting the voltage-gated calcium channels (VGCC; Yen-Patton et al., 1988; Li et al., 2014; Peng et al., 2021). The effects of real-time EMP radiation on neuron activity might be mediated by the interrupted VGCC by EMPs, but the underlying mechanisms were far from being clarified. Different from the above changes, the neuron activity in hippocampal CA1 was significantly inhibited on day 1 and day 7 after EMP exposure, when mice entered the central area of the open field, or contacted the novel object in the NOE experiment. And we speculated that the inhibited responses of the hippocampal neurons to the novelty caused by EMPs, as described in Figure 6; were the important reasons explaining the impaired spatial learning and memory ability in mice when conducting Morris water maze and novel arm discrimination tasks. Studies have shown that EMP radiation could cause synapse connection damages, oxidative stress (Deng et al., 2014; Jiang et al., 2016), inflammatory responses (Zhang et al., 2019, 2019), which might be mechanisms underlying the inhibited neuronal functions after EMP radiation.

## Conclusions

EMP exposure impaired the spatial learning and memory ability and disturbed the neuronal activities in hippocampal CA1 in mice. The disturbed calcium activities in hippocampal CA1 neurons constituted the cytological mechanisms of the impaired spatial learning and memory ability caused by EMPs. However, the mechanisms how EMPs affect neuronal calcium activities requires to be further studied.

## Data Availability Statement

The raw data supporting the conclusions of this article will be made available by the authors, without undue reservation.

## Ethics Statement

The animal study was reviewed and approved by The Institutional Animal Care and Use Committee of the Beijing Institute of Radiation Medicine.

## Author Contributions

YL and HZ: conceptualization, methodology, writing—reviewing and editing. YH, WL, and ZX: methodology, software, data curation, writing—original draft preparation. XJ, YY, CY, and CH: methodology. All authors contributed to the article and approved the submitted version.

## Conflict of Interest

The authors declare that the research was conducted in the absence of any commercial or financial relationships that could be construed as a potential conflict of interest.

## Publisher’s Note

All claims expressed in this article are solely those of the authors and do not necessarily represent those of their affiliated organizations, or those of the publisher, the editors and the reviewers. Any product that may be evaluated in this article, or claim that may be made by its manufacturer, is not guaranteed or endorsed by the publisher.

## Supplementary Material

Supplementary Figure 1 Fiber photometry on GFP-expressing mice, and GCaMP-expressing mice treated by sham exposure. **(A)** The △F/F_0_(%) of GFP expressed in hippocampal CA1 area recorded by fiber photometry. 0 s for △F/F0, marked by dotted lines, indicated that EMP exposure starting up, or mice entering central area in an open field (OF), or exploring a novelty during novel object exploration test (NOE). **(B,C)** Fiber photometry on GCaMP-expressing mice treated by sham exposure when seeking a novelty (*n* = 3 mice). **(B)** The combined line diagram of calcium amplitude △F/F_0_ (%) in CA1 neurons before, and day 1, day 7 after sham exposure. 0 s for △F/F0, marked by dotted lines, indicated that mice were exploring a novelty (NOE). **(C)** Plot of AUC in multiple trials from three biological replicates before and after sham exposure. The data are presented as mean ± SEM. One-way ANOVA followed by Bonferroni’s *post-hoc* tests were performed to evaluate the differences between multiple groups in **(C)**.
