# Supplementary material for: High-Power Electromagnetic Pulse Exposure of Healthy Mice: Assessment of Effects on Mice Cognitions, Neuronal Activities, and Hippocampal Structures
Source: Front Cell Neurosci. 2022 Jun 29;16:898164. doi: 10.3389/fncel.2022.898164 (PMC9374008; doi:10.3389/fncel.2022.898164)
Supplement: Supplementary Figure 1 — Fiber photometry on GFP-expressing mice, and GCaMP-expressing mice treated by sham exposure. (A) The △F/F0(%) of GFP expressed in hippocampal CA1 area recorded by fiber photometry. 0 s for △F/F0, marked by dotted lines, indicated that EMP exposure starting up, or mice entering central area in an open field (OF), or exploring a novelty during novel object exploration test (NOE). (B,C) Fiber photometry on GCaMP-expressing mice treated by sham exposure when seeking a novelty (n = 3 mice). (B) The combined line diagram of calcium amplitude △F/F0 (%) in CA1 neurons before, and day 1, day 7 after sham exposure. 0 s for △F/F0, marked by dotted lines, indicated that mice were exploring a novelty (NOE). (C) Plot of AUC in multiple trials from three biological replicates before and after sham exposure. The data are presented as mean ± SEM. One-way ANOVA followed by Bonferroni’s post-hoc tests were performed to evaluate the differences between multiple groups in (C). [file Image_1.pdf]

Supplementary

The highest fluorescence amplitude $\Delta F/F_0$ (%) of GFP expressed in the hippocampal CA1 area was less than 0.5% when mice receiving EMP exposure, or entering the central area in an open field, or exploring a novelty (Figure 1A). The GCaMP signals of hippocampus CA1 neurons of the sham-exposed mice when exploring a novelty showed no significant differences before and after sham exposure, suggesting that the changes of the GCaMP signals in the EMP-exposed group were attributed to the EMP radiation (Figure 1B, C).

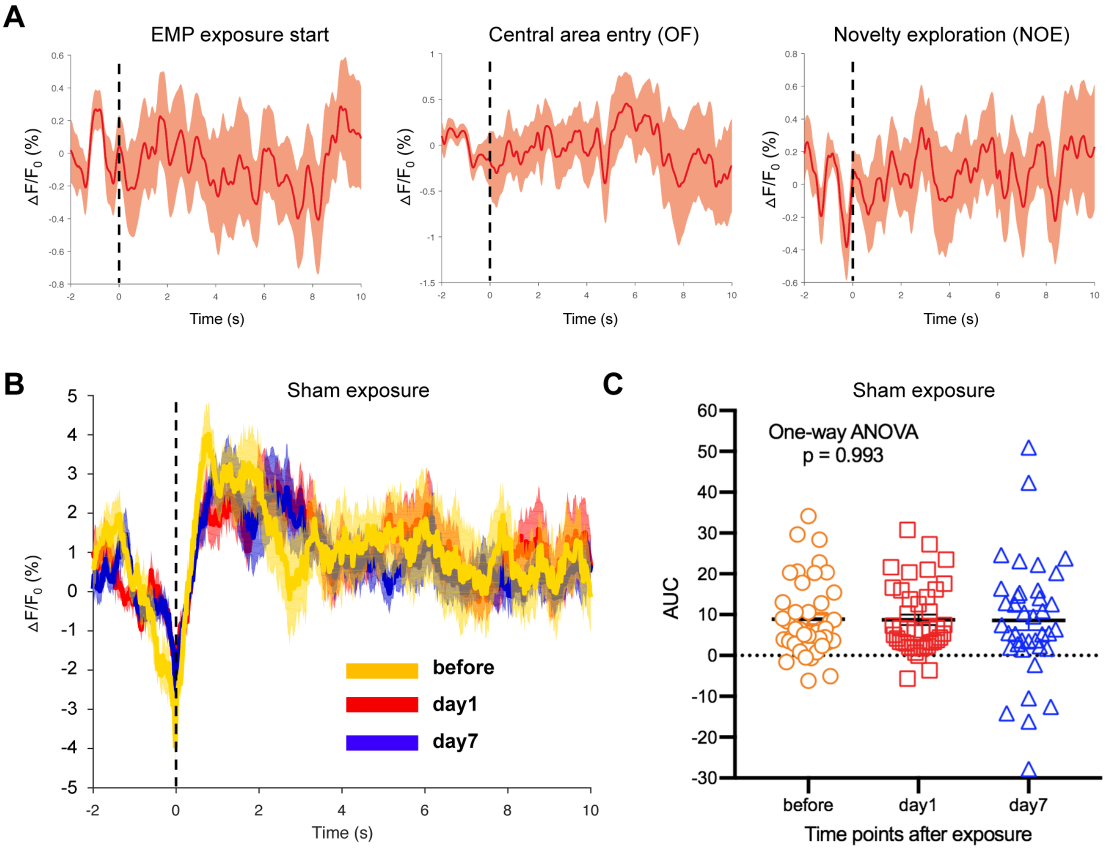

Figure 1 Fiber photometry on GFP-expressing mice, and GCaMP-expressing mice treated by sham exposure. **(A)** The $\Delta F/F_0$ (%) of GFP expressed in hippocampal CA1 area recorded by fiber photometry. 0 s for ΔF/F0, marked by dotted lines, indicated that EMP exposure starting up, or mice entering central area in an open field (OF), or exploring a novelty during novel object exploration test (NOE). **(B-C)** Fiber photometry on GCaMP-expressing mice treated by sham exposure when seeking a novelty (n = 3 mice). **(B)** The combined line diagram of calcium amplitude $\Delta F/F_0$ (%) in CA1 neurons before, and day 1, day 7 after sham exposure. 0 s for ΔF/F0, marked by dotted lines, indicated that mice were exploring a novelty (NOE). **(C)** Plot of AUC in multiple trials from three biological replicates before and after sham exposure. The

data are presented as mean ± SEM. One-way ANOVA followed by Bonferroni's post-hoc tests were performed to evaluate the differences between multiple groups in **(C)**.
